# Evaluation of Laboratory Toxicities and Field Application of Plant Spray Oil and Its Mixture with *Metarhizium anisopliae* Against *Diaphorina citri Kuwayama* (Hemiptera: Liviidae)

**DOI:** 10.3390/insects16070663

**Published:** 2025-06-25

**Authors:** Dasong Chen, Jiaqi Suo, Jianquan Yan, Lijia Chen, Fenghao Chen, Jianying Huang, Haitao Duan, Gecheng Ouyang, Xiang Meng

**Affiliations:** 1 Guangdong Key Laboratory of Animal Conservation and Resource Utilization, Guangdong Public Laboratory of Wild Animal Conservation and Utilization, Institute of Zoology, Guangdong Academy of Science, Guangzhou 510260, China; chendasong@gmail.com (D.C.);; 2 College of Plant Protection, South China Agricultural University, Guangzhou 510642, China; 3 College of Life Sciences, Guangzhou University, Guangzhou 510006, China; 4 Qingyuan Agricultural Science and Technology Extension Service Center, Qingyuan 511518, China; pinganyi@foxmail.com; 5 Fogang Chutou Baba Ecological Agriculture Co., Ltd., Qingyuan 511600, China

**Keywords:** plant spray oil, *Metarhizium anisopliae*, *Diaphorina citri*, virulence, synergistic effect, mix proportion, chemical substitute

## Abstract

Overusing chemicals to manage *Diaphorina citri* has created insecticide resistance and negative impacts on the natural ecosystem. This has prompted the need to develop new methods of control. In the present study, we evaluated the toxicity of plant spray oil, *Metarhizium anisopliae*, and their combined formulations against *D. citri* adults through laboratory bioassays. We tested varying concentrations and application doses of the individual agents and their mixtures to determine synergistic effects. The optimal mixing ratio of *M. anisopliae* and plant spray oil for enhanced pest control efficacy was also identified. The results showed that the control effect of plant spray oil and *M. anisopliae* on *D. citri* became more pronounced as the concentration increased. When the two were mixed in different proportions, the synergistic effect of plant spray oil was the strongest when the mixing ratio was 5:5. Field trials demonstrated that a combined treatment of plant spray oil emulsion (9.10 g/L) and *M. anisopliae* (1 × 10^8^ spores/mL) achieved over 80% relative control efficacy against adult *D. citri* by the 6th day post-treatment. Its efficacy further increased to 96.28% by the 8th day, demonstrating its potential to replace chemical control methods. This study provides a practical example for exploring biopesticides and leveraging the synergistic effects of biogenic pesticides in preventing and controlling pests.

## Simple Summary

*Diaphorina citri*, the natural transmission vector of the destructive disease Huanglongbing, is the most important pest in citrus production. This research provides a method for the eco-friendly control of *D. citri*. A new plant spray oil, based on plant glycerides extracted from soy sauce waste residue, was developed into an environmentally friendly pesticide, which can quickly and effectively control *D. citri*. When mixed with *Metarhizium anisopliae*, it had a significant synergistic effect. Field application showed that the control efficacy of the plant spray oil and *M. anisopliae* mixture on *D. citri* was equivalent to that of chemical control, presenting a viable alternative for eco-friendly pest management. This study provides a scientific basis for effectively controlling *D. citri* and preventing the further spread of Huanglongbing in the field.

## 1. Introduction

The Asian citrus psyllid (ACP), *Diaphorina citri* Kuwayama (Hemiptera: Psyllidae), is a natural vector insect that transmits Huanglongbing (HLB), the most devastating disease of citrus worldwide [1,2,3]. To date, the primary management measure for *D. citri* has been chemical control by using insecticides [4,5]. It is well-known that excessive insecticide applications may result in negative consequences such as environmental pollution [6], the development of insecticide resistance [7,8], and negative impacts on natural enemy populations [9]. Therefore, to mitigate the adverse effects of insecticides, the use of eco-friendly control agents, such as botanical pesticides and entomopathogenic fungi (EPFs), may be a better strategy for Integrated Pest Management (IPM) [10].

Over more than a century, EPFs have become one of the most critical tools in biological pest control due to their strong pathogenicity, diverse host range, and environmental friendliness [11,12]. Among EPFs, *Metarhizium anisopliae* (Hypocreales: Clavicipitaceae) is well-known and reported to infect many sucking pests [13,14]. However, the field application of *M. anisopliae* has received limited attention due to its slower action compared to conventional methods in achieving sufficient insect mortality, and spore germination is adversely affected by ultraviolet radiation under natural conditions [15]. Recently, many studies have explored different strategies to enhance the virulence of these fungi, such as the expression of Bt toxin proteins in *Beauveria bassiana*, in applications with natural enemies, synthetic oils, and chemical pesticides [16,17].

Plant spray oil is derived from plant glycerol esters extracted from plant sauce waste and formulated into a bio-derived pesticide through specialized processing [18]. Its insecticidal property is mainly contact-based, which forms an oil film over the surface of the insect body and seals off the stomata, egg stomata, or receptors of the insect, leading to asphyxiation. Moreover, plant spray oil also acts as a pest repellent, interfering with critical behaviors such as host positioning, egg laying, and other behaviors [18]. In addition to its insecticidal effects, plant spray oil can enhance the microenvironment for fungal activity, thereby protecting and boosting the pest lethality of fungal spores [19,20]. However, the combined application of plant spray oil with *M. anisopliae* for the control of *D. citri* has not yet been explored.

In this study, we investigated the toxicity of plant spray oil, *M. anisopliae*, and their mixtures against *D. citri*. The optimal proportion with synergism was screened by a cross-assay and co-toxicity factor method. This study not only provides a technical reference for the green control of *D. citri* but also provides practical guidance for the field control of *D. citri*. The experimental results can be further validated in the field.

## 2. Materials and Methods

### 2.1. Insects

Adults of *D. citri* were collected from *Murraya paniculata* (L.) Jack (Sapindales: Rutaceae) (which is *D. citri*’s favorite host) at the Sun Yat-sen University (Lat: 23.0966° N, Lng: 113.2986° E), and then they were reared together with a *M. paniculata* plant on a multi-tier plant cultivation shelf in an artificial isolated climate room with a temperature of 26 ± 1 °C, a relative humidity of 75 ± 5%, and a photoperiod of 14 h:10 h (L:D). Prior to experimentation, the insect colony was maintained for more than three successive generations.

### 2.2. Plant Spray Oil

The plant spray oil consisted of 93% plant glyceride emulsion, generously provided by Guangzhou European Union Biotechnology Company Ltd. (Guangzhou, China). The plant glyceride emulsion was dissolved with deionized water to prepare a 50-fold dilution solution of plant spray oil emulsion (18.2 g/L; *v*/*v* = 1:50). Lower doses of plant spray oil (9.10, 4.55, 2.28, and 1.14 g/L; *v*/*v* = 1:100, 1:200, 1:400, 1:800) were obtained through a series dilution method for subsequent experiments.

### 2.3. Metarhizium anisopliae Culture

*Metarhizium anisopliae* was isolated from the corpse of *D. citri*, which was naturally infected, and obtained through strain identification and optimization. The strain was stored at the Environmental Insect Research Center of the Institute of Zoology, Guangdong Academy of Sciences. The strain was cultured in Petri dishes (9 cm dia.) that contained PDA medium and incubated at 26 ± 1 °C for 7 days in darkness. After complete sporulation, spores were collected from the plates.

The fungal conidia were suspended in sterile deionized water containing 0.2% Tween-80 solution. The conidia were counted by using a hematology analyzer under a compound microscope. Then, the conidial suspension was adjusted to 2 × 10^8^, 1 × 10^8^, 5 × 10^7^, and 2.5 × 10^7^ conidia/mL suspension.

### 2.4. Bioassay

#### 2.4.1. Toxicity of Plant Spray Oil to *Diaphorina citri*

Two sets of parallel experiments with a high dose of 10 mL and a low dose of 5 mL were conducted on the adults of *D. citri* using an identical methodology. Fresh *M. paniculata* tender shoots were selected and placed in a net bag (25 cm × 30 cm), with each bag containing 20 adults of *D. citri* that were at the same growth period and activity. A hand-held sprayer was used to evenly spray plant spray oil and deionized water at five different concentration gradients onto the surface of the net bags. Following the spraying, the bags were placed in a light incubator set at 26 ± 1 °C with a relative humidity of 75 ± 5% and a photoperiod of 12 L:12 D. We utilized 0.02% Tween-80 sterile water as a control treatment. Adults of *D. citri* were randomly collected and subjected to three independent biological replications on distinct plants. The number of deaths was recorded at 3 and 6 h after treatment, and the corrected mortality and virulence were calculated.

#### 2.4.2. Toxicity of *Metarhizium anisopliae* to *Diaphorina citri*

The spray application method was consistent with that described in Section 2.4.1. Following 10 mL spraying, *D. citri* adults were placed on fresh *M. paniculata* tender shoots in a light incubator at 26 ± 1 °C, 75 ± 5% RH, and 12 L:12 D for feeding. We utilized 0.02% Tween-80 sterile water as the blank control. Each treatment was replicated three times, with 20 random healthy adults per replicate on distinct plants. Post-experimental plants were relocated from the trial zone and surface-sterilized via solar exposure in outdoor conditions. Adult mortality was examined at 3 h, 6 h, 1 d, 2 d, and 3 d after treatment.

#### 2.4.3. Toxicity of the Mixed Plant Spray Oil and *Metarhizium anisopliae* to Adults of *Diaphorina citri*

According to the LC_50_ and LT_50_ values derived from the tests in Section 2.4.1 and Section 2.4.2, appropriate concentrations of plant spray oil and *M. anisopliae* were prepared. Eleven concentration gradients were set up according to different volume ratios, and the proportions of the mixed solutions were as follows: plant spray oil/*M. anisopliae* = 10:0, 9:1, 8:2, 7:3, 6:4, 5:5, 4:6, 3:7, 2:8, 1:9, and 0:10. A blank control consisting of 0.02% Tween-80 sterile water was also included. Each treatment was replicated three times, with 20 adults in each replicate on independent plants to avoid cross-influence. The plants were removed from the test area after experimentation and exposed to outdoor sunlight for sterilization. The spraying method was the same as that in Section 2.4.1. Following 10 mL spraying, fresh *M. paniculata* tender shoots were transferred into a light incubator at 26 ± 1 °C, 75 ± 5% RH, and 12 L:12 D for 26 h incubation. Subsequently, the interactive test method and co-toxicity factor analysis were employed for detection and analysis.

#### 2.4.4. Field Control Efficacy of Plant Spray Oil and *Metarhizium anisopliae* Against *Diaphorina citri*

The field efficacy trial was conducted targeting three-year-old lemon trees in a lemon orchard spanning 33.33 hectares of Cutou Baba Ecological Agriculture Co., Ltd. in Fogang County, Guangdong Province. The absence of insecticide or fertilizer application prevented any interference with the field trials. The field trial encompassed 4 treatments: A: a control group receiving no intervention; B: 0.5 × 10^8^ conidia/mL *M. anisopliae* + 4.55 g/L plant spray oil; C: 1 × 10^8^ conidia/mL *M. anisopliae* + 9.10 g/L plant spray oil; D: orchard conventional chemical treatment. Each treatment was replicated three times, which involved five trees per replication, with spraying twice within 10 days during the shoot growth period. Uniform application was ensured across all treatments utilizing a consistent-frequency sprayer, and orchard management was homogeneously implemented for every treatment group.

A five-point sampling method was employed to investigate the population of *D. citri* adults per replication. Initial surveys were conducted on the day before treatment, with subsequent assessments on the 2nd, 4th, 6th, and 8th day after treatment. According to the statistical results, the population density, population reduction rate, and relative control efficiency were calculated.

### 2.5. Data Analysis

The experimental data were statistically analyzed using SPSS 19.0 software. The mortality rate and corrected mortality rate were calculated. The virulence regression equation, LC_50_, LT_50_, co-toxicity coefficient, and 95% confidence limits were determined. One-way analysis of variance was used to analyze the experimental data of the decreasing rate of the pest population, relative control efficacy, mortality rate, and corrected mortality rate. And the mean differences between the treatments were tested by ANOVA at a 5% level of significance. Multi-factor analysis of variance was used to determine the influence of plant spray oil, *M. anisopliae*, and exposure time on the mortality rate of adult *D. citri*. The calculation formulas are as follows:

The mortality rate was used to calculate the proportion or percentage of pests in a trial that die within a specific time period:

Mortality rate = number of dead insects/number of tested insects × 100%

The corrected mortality was used to calculate the statistical correction to the test mortality rate with the control mortality rate:

Adjusted mortality = (treatment group mortality rate − control group mortality rate)/(1 − control group mortality rate) × 100%

The co-toxicity coefficient (CTF) was used to evaluate the interactive effects (e.g., synergism, antagonism, or additive effects) of two or more combined agents:

Co-toxicity coefficient = (actual mortality − theoretical mortality)/theoretical mortality × 100%

The decreasing rate measured the treatment’s field efficacy in pest populations:

Decreasing rate of pest = (number of treatment group insects before treatment − number of treatment group insects after treatment)/number of treatment group insects before treatment × 100%

Relative control efficacy is a standardized metric used to evaluate the effectiveness of a pest control treatment by comparing changes in pest populations between treated and control groups, while accounting for natural population fluctuations:

Relative control efficacy = (treatment decreasing rate − contral decreasing rate)/(1 − contral decreasing rate) × 100%

## 3. Results

### 3.1. Toxicity of Plant Spray Oil to Diaphorina citri

Our results showed that different concentrations and doses of plant spray oil significantly affected the corrected mortality rates (%) of *D. citri* compared to the control (Table 1). After plant spray oil treatment, the corrected mortality rate of the highest mass concentration was 83.33 ± 6.01% (5 mL)~100 ± 0.00% (10 mL). Toxicity measurements showed that the LC_50_ of *D. citri* decreased with the escalating concentrations of plant spray oil and that lethal activity decreased at 6 h compared to 3 h.

### 3.2. Toxicity of Metarhizium anisopliae to Diaphorina citri

**Figure 1:**
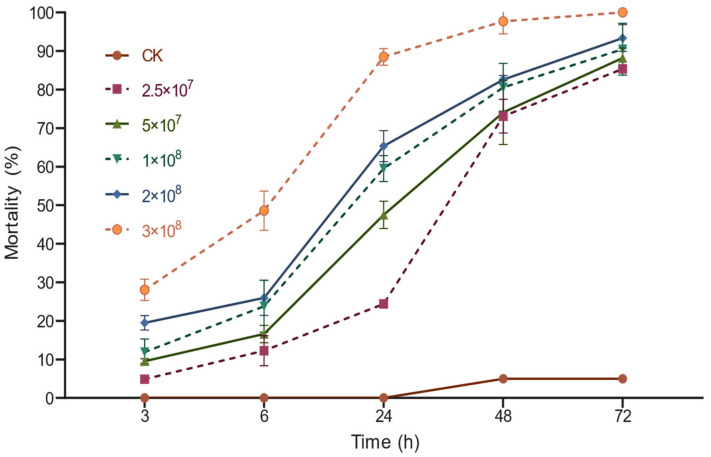
Comparison of time–mortality relationship for *M. anisopliae* used on *D. citri* adults inoculated with spores at varied concentrations. Spore suspensions were prepared in 0.02% Tween-80 solution at concentrations of 2.5 × 10^7^, 5 × 10^7^, 1 × 10^8^, 2 × 10^8^, and 3 × 10^8^ spores/mL, with the 0.02% Tween-80 solution as the control.

The toxicity of different concentrations of *M. anisopliae* spore suspensions to *D. citri* adults showed that the cumulative mortality of *D. citri* adults escalated in tandem with both the rising *M. anisopliae* spore concentration and the prolonged duration of spore exposure (Figure 1). Within the consistent treatment duration, a decline in the cumulative mortality of *D. citri* was observed as the spore concentration of *M. anisopliae* decreased (Table 2). Optimal insecticidal outcomes were achieved at the top spore concentration (4 × 10^8^ spores/mL). The mortality rate was significantly higher than that in the blank control group on the first day. By the 2nd day, the mortality and corrected mortality of adults were higher than 90% (Table 2). The control efficacy at a spore concentration of 2.5 × 10^7^ spores/mL against adult *D. citri* was minimal on the first day. However, the insecticidal impact would be progressively amplified over time, culminating in a mortality rate that markedly transcended that of the control group after 2 days.

### 3.3. Toxicity of the Mixed Plant Spray Oil and Metarhizium anisopliae to Adults of Diaphorina citri

**Figure 2:**
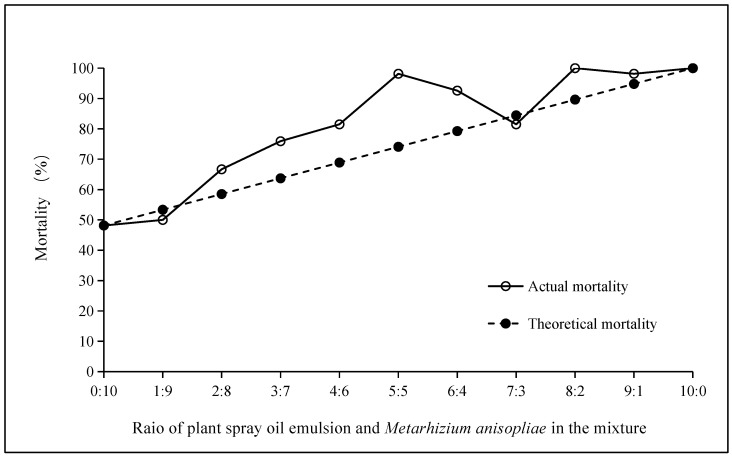
Synergistic effect of plant spray oil and *M. anisopliae* on adults of *D. citri* according to interactive determination method.

The synergistic toxicity of the mixture of plant spray oil and *M. anisopliae* in different proportions against the adults of *D. citri* was evaluated by an interactive test method. The results at the 6th hour after treatment are depicted in Figure 2. When the mixing ratios of plant spray oil and *M. anisopliae* were 9:1, 1:9, and 7:3, the actual mortality rates of *D. citri* closely adhered to the theoretical equivalent line, indicating an additive interaction under these ratios. At the mixing ratios of 2:8, 3:7, 4:6, 5:5, 6:4, and 8:2, the actual mortality rate of *D. citri* surpassed the theoretical equivalent line, indicating that the mixing ratios of the two pesticides had a robust synergistic effect on the control of *D. citri*; the longer the vertical line is, the stronger the synergy is. That is, the synergy is 2:8, 8:2, 3:7, 4:6, 6:4, and 5:5 from small to large.

The toxicity of the mixtures with different proportions to the adults of *D. citri* was analyzed by the co-toxicity factor method. The results are shown in Table 3 (after 26 h of treatment). When the mixed proportions of plant spray oil and *M. anisopliae* were 1:9, 2:8, 3:7, 4:6, 6:4, 7:3, 8:2, and 9:1, the co-toxicity factors were between −20 and 20, indicating that there was an additive effect between the two. When the mixing ratio was 5:5, the co-toxicity factor was 32.5, which is greater than 20, indicating that the two agents have a synergistic effect when mixed according to this ratio. The largest ratio of co-toxic factors was 5:5, which was consistent with the results of the cross-assay.

### 3.4. Field Control Efficacy for Diaphorina citri

**Figure 3:**
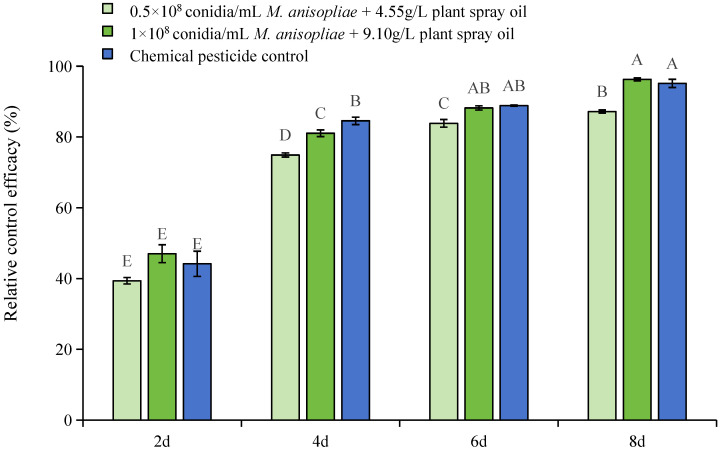
Field control efficacy of *D. citri* with different treatments in the field. The experimental data were analyzed using one-way ANOVA, and Duncan’s new multiple range test was employed to test the differences among treatments. Different letters indicate significant differences (*p* < 0.05). Error bars represent the standard error of the mean.

No significant differences in the insect density of *D. citri* in each experimental area were observed before application. After application, the control efficacy of plant spray oil and *M. anisopliae* in mitigating *D. citri* populations was markedly superior to that of the control group, and was comparable to that of the chemical control group (2 d, F = 65.70, *p* < 0.001; 4 d, F = 332.08, *p* < 0.001; 6 d, F = 343.83, *p* < 0.001; 8 d, F = 641.55, *p* < 0.001). The potency of the mixture amplified in tandem with the increase in the concentration, exerting a sustained suppressive influence on the demographic expansion of *D. citri* (Figure 3). Further analysis showed that the relative control efficacy and decreasing rate of pests when using the plant spray oil and *M. anisopliae* mixture significantly transcended those of the control group (8 d relative control efficacy, F = 93.30, *p* < 0.001; 8 d decreasing rate of pests, F = 322.20, *p* < 0.001). Among the treatments, the 8-day post-application spray comprising 1 × 10^8^ conidia/mL *M. anisopliae* + 9.10 g/L plant spray oil yielded the most outstanding control efficacy, reaching 96.28%.

## 4. Discussion

The employment of eco-friendly and safe pesticides to control plant pests not only safeguards the security of food production but also mitigates pesticide residues and environmental pollution. Moreover, it facilitates the utilization and exploration of the synergism among new green pesticides, thereby offering novel avenues for the prevention and control of field pests. Both plant spray oil and *M. anisopliae* are widely utilized as green, pollution-free insecticides in agricultural production, but research gaps persist regarding whether their combined application can enhance efficacy against *D. citri*.

Plant oil derived from fermented foods represents a novel category of plant oils distinguished by its facile environmental degradation, cost-effective production, safety for natural predators, and inability to provoke pest resistance [18]. It plays a pivotal role in the prevention and control of small pests and some foliar microbial diseases. For example, castor oil has been effectively employed to control larvae of *Plutella xylostella* (Lepidoptera: Plutellidae) in the field, concurrently deterring adults from spawning [21]. The results of our experiments show that plant spray oil can be effectively harnessed against citrus planthopper adults, with its lethal impact on *D. citri* adults intensifying in tandem with escalating concentrations and dosages of plant spray oil. Consequently, the adoption of high-dose and low-concentration applications of plant spray oil in the field is more in line with the principles of sustainable pest management.

To date, many EPFs have been reported for the control of *D. citri*, including *B. bassiana*, *M. anisopliae*, *Aspergillus fijiensis*, *Paecilomyces variotii*, *Hirsutella citriformis*, and *Akanthomyces lecanii* [22,23,24,25,26]. Among them, *M. anisopliae*, which has been commercially produced and applied, is a prominent entomopathogenic fungus widely distributed in nature. It is well-established that *M. anisopliae* has already been effective against over 200 insect species, encompassing *Ceratitis capitata* Wiedemann (Diptera: Tephritidae) [27], *Spodoptera littoralis* (Lepidoptera: Noctuidae) [28], *Spodoptera exigua* (Lepidoptera: Noctuidae) [29], and *Aphis gossypii* Glover (Hemiptera: Aphididae) [30], among others. Our results showed that the control effect of *M. anisopliae* on adults of *D. citri* was noticeable (89.98%, 92.90%, and 100% mortality rates with 1 × 10^8^, 2 × 10^8^, and 3 × 10^8^ conidia/mL on 3rd day). The efficacy in controlling adults of *D. citri* is superior to that in previously reported studies, which also found that M. anisopliae isolates caused a severe infection in *D. citri* (60% with 1 × 10^8^ conidia/mL) [31]. Nevertheless, the common problem faced by our research is that when *M. anisopliae* was used alone, its effect manifested gradually over a relatively prolonged period, the initial mortality rate was modest, and it had environmental sensitivity [32,33,34].

Plant oil not only exhibits a contact and repellent effect on pests but also can be synergistically combined with other control factors. Plant oil can serve as an adjuvant to facilitate the dispersion, wetting, and deposition of other chemical pesticides on the surface of organisms, thereby enhancing the penetration and coverage of pesticides on the plant surface [18]. This significantly boosts efficiency while decreasing chemical pesticide dosage. In this study, the combination of *M. anisopliae* and plant spray oil at a 5:5 ratio showed the highest toxicity to adult *D. citri*. In theory, the mixture ratio of 5:5 can significantly enhance the efficacy while decreasing the dosage requirement of *M. anisopliae*. Some studies reported that EPFs achieved control at percentages of 35–60% in nymphs and 22–50% in adults [29]. Our trials demonstrate that application comprising 1 × 10^8^ conidia/mL *M. anisopliae* + 9.10 g/L plant spray oil yielded outstanding control efficacy surpassing 95%. This control efficacy can drive farmers’ willingness to substitute chemical pesticides with fungal insecticides.

Green pest control, an emergent concept put forward in recent years, inclusively leverages diverse methodologies and green pesticides to control the occurrence of diseases and pests. However, practical application has revealed that most green pesticides exhibit modest insecticidal efficacy, which restricts their popularization. Exploring the synergistic interaction among green pesticides and enhancing their insecticidal efficacy are pivotal for facilitating their utilization and popularization. The results of this study play an important guiding role in conforming to the development trend of green agriculture and pollution-free agriculture, giving full play to the synergistic effect of microbe and organic spray oil, and mitigating pesticide usage. Furthermore, plant oil and *M. anisopliae* are greatly affected by environmental factors such as temperature and humidity, leading to inconsistent control effects and durations in the field. Therefore, the toxicity of the combination of plant spray oil and *M. anisopliae* to *D. citri* needs to be verified in additional field trials to support the application of the mixture of plant spray oil and *M. anisopliae* in the control of *D. citri*.

## Figures and Tables

**Table 1:** Toxicity of different doses of plant spray oil to *D. citri* adults.

Dose	Mass Concentration g/L	Corrected Mortality/%	Regression Equation	LC_50_ (g/L)	95% Confidence Interval	χ^2^
5 mL (6 h)	1.14	6.67 ± 4.41 d	Y = 0.138X − 1.182	8.561	6.526–11.158	4.699
	2.28	10.00 ± 0.00 d				
	4.55	30.00 ± 5.00 c				
	9.10	65.00 ± 0.00 b				
	18.2	83.33 ± 6.01 a				
10 mL (3 h)	1.14	13.56 ± 2.94 e	Y = 0.264X − 1.491	5.638	4.448–7.292	0.996
	2.28	22.03 ± 3.40 d				
	4.55	32.20 ± 3.40 c				
	9.10	88.14 ± 1.69 b				
	18.2	100.00 ± 0.00 a				

Data are mean ± SE. Data from same experiment followed by different letters indicate significant difference at 5% level by Duncan’s new multiple range method. Statistical analyses were performed on each condition separately.

**Table 2:** Toxicity of different concentrations of *M. anisopliae* to *D. citri* adults.

Concentrations (×10^7^ conidia/mL)	Corrected Mortality/% (1 d)	LC_50_ (×10^7^ conidia/mL)	Corrected Mortality/% (3 d)	LT_50_/h	Regression Equation	95% Confidence Interval	χ^2^
40	88.48 ± 2.15 a	10.082	100 ± 0 a	10.660	Y = 0.063X − 0.550	4.854–16.266	2.227
20	64.36 ± 5.41 b		92.90 ± 3.49 ab	21.453	Y = 0.036X − 0.777	13.331–29.036	2.453
10	59.52 ± 3.37 b		89.98 ± 7.09 b	26.070	Y = 0.035X − 0.921	17.737–34.384	3.186
5	47.50 ± 3.56 c		87.56 ± 2.70 b	32.342	Y = 0.035X − 1.138	24.289–41.171	1.750
2.5	24.41 ± 0.84 d		84.59 ± 0.53 b	39.209	Y = 0.039X − 1.527	31.606–48.133	1.88

Data are mean ± SE. Data from same experiment followed by different letters indicate significant difference at 5% level by Duncan’s new multiple range method.

**Table 3:** Synergistic effect of plant spray oil and *M. anisopliae* on adults of *D. citri* according to co-toxicity factor (CTF) method.

Plant Spray Oil/*M. anisopliae*	Actual Mortality	Theoretical Mortality	CTF
0:10	48.148	48.148	0
1:9	50.000	53.333	−6.249
2:8	66.667	58.518	13.926
3:7	75.926	63.703	19.188
4:6	81.481	68.889	18.279
5:5	98.148	74.074	32.500
6:4	92.593	79.259	16.823
7:3	81.481	84.444	−3.509
8:2	100.000	89.630	11.570
9:1	98.148	94.815	3.515
10:0	100.000	100.000	0

Note: CTF: co-toxicity coefficient; CTF > 20: synergistic interaction; CTF < −20: antagonistic interaction; −20 ≤ CTF ≤ 20: additive interaction.

## Data Availability

The raw data supporting the conclusions of this article will be made available by the authors on request.
